# Lipocalin 2 receptors: facts, fictions, and myths

**DOI:** 10.3389/fimmu.2023.1229885

**Published:** 2023-08-11

**Authors:** Sarah K. Schröder, Natalie Gasterich, Sabine Weiskirchen, Ralf Weiskirchen

**Affiliations:** ^1^ Institute of Molecular Pathobiochemistry, Experimental Gene Therapy and Clinical Chemistry (IFMPEGKC), RWTH University Hospital Aachen, Aachen, Germany; ^2^ Institute of Neuroanatomy, RWTH University Hospital Aachen, Aachen, Germany

**Keywords:** NGALR, SLC22A17, Megalin, LRP2, MC4R, inflammation, iron

## Abstract

The human 25-kDa Lipocalin 2 (LCN2) was first identified and purified as a protein that in part is associated with gelatinase from neutrophils. This protein shows a high degree of sequence similarity with the deduced sequences of rat α_2_-microglobulin-related protein and the mouse protein 24p3. Based on its typical lipocalin fold, which consists of an eight-stranded, anti-parallel, symmetrical β-barrel fold structure it was initially thought that LCN2 is a circulating protein functioning as a transporter of small lipophilic molecules. However, studies in *Lcn2* null mice have shown that LCN2 has bacteriostatic properties and plays a key role in innate immunity by sequestering bacterial iron siderophores. Numerous reports have further shown that LCN2 is involved in the control of cell differentiation, energy expenditure, cell death, chemotaxis, cell migration, and many other biological processes. In addition, important roles for LCN2 in health and disease have been identified in *Lcn2* null mice and multiple molecular pathways required for regulation of *Lcn2* expression have been identified. Nevertheless, although six putative receptors for LCN2 have been proposed, there is a fundamental lack in understanding of how these cell-surface receptors transmit and amplify LCN2 to the cell. In the present review we summarize the current knowledge on LCN2 receptors and discuss inconsistencies, misinterpretations and false assumptions in the understanding of these potential LCN2 receptors.

## 1 Introduction

Lipocalin 2 (LCN2; OMIM:600181), also known as neutrophil gelatinase-associated lipocalin (NGAL), siderocalin (Scn), oncogenic lipocalin 24p3, super inducible protein 24 (SIP24), uterocalin, α_2_-microglobulin-related protein, and neu-related lipocalin (NRL) is a 25-kDa protein involved with inflammatory response in multiple diseases. Historically, the first amino acid sequence of a LCN2 orthologue was deduced from a cDNA sequence of rat, which was isolated fortuitously whilst screening a library with a synthetic oligonucleotides designed to a sequence located in the ribosomal protein P1 (1). Based on its high degree of similarity with α_2_ microglobulin, rat LCN2 was initially termed α_2_-microglobulin-related protein (1). The identification of mouse LCN2 was reported one year later by Hraba-Renevey and colleagues (2). In their study they isolated mouse *Lcn2* mRNA by differential screening as a gene that is induced by SV40 in G_o_-arrested primary mouse kidney cell cultures. The name 24p3 of this gene was given in a very pragmatic way and simply referred to the plate (no. 24) and the sequential assigned number of the cDNA sequence (no. 3) isolated from respective plate (Suzanne Hraba-Renevey, personal communication).

**Figure 1:**
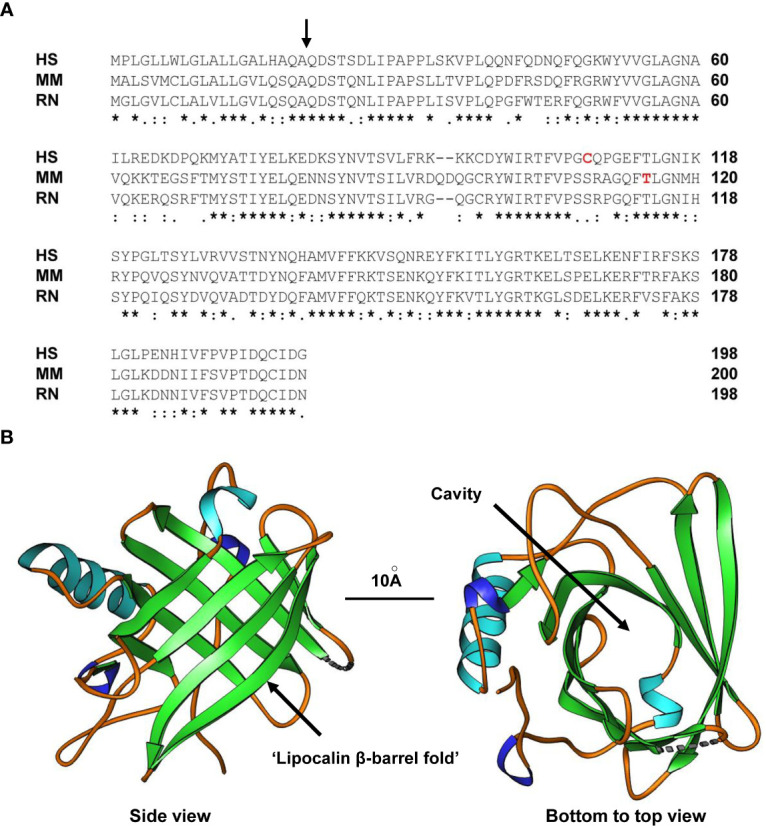
Structure of LCN2. **(A)** Alignment of human (HS), mouse (MM) and rat (RN) LCN2 proteins. The side of cleavage of the secretory signal peptide is marked by a black arrow. In addition, the tyrosine in mouse LCN2 (T115) shown to be a PKCδ phosphorylation site and the cysteine in human LCN2 (C107) shown to form the disulfide bridge with human MMP-9 is marked in red letters. An asterisk (*) indicates fully conserved residues, a colon (:) conservation between groups of strongly similar properties, and a period (.) conservation between groups of weakly similar properties. Gaps in the aligned sequences are indicated by dashes. **(B)** Side (*left*) and bottom-to-top (*right*) views of human LCN2 showing the typical eight-stranded, anti-parallel, symmetrical β-barrel fold that is characteristic of members of the lipocalin family. The formed cavity is supposed to permit binding of a broad array of bulky ligands. The depicted structure of the apo-form of human LCN2 determined by X-ray diffraction resolved at resolution of 2Å was generated using the Ribbons XP software (version 3.0) and structure coordinates deposited in the RCSB Protein Data Bank (4) [http://www.rcsb.org] under accession no. 3BX8. A size marker (10Å) is given.

Subsequently, human LCN2 was identified another four years later and purified to apparent homogeneity from exocytosed material of phorbol myristate acetate-stimulated neutrophils and suggested to be a new member of the lipocalin family (3). This family includes small secreted proteins that share a characteristic three-dimensional fold comprised of a single eight-stranded continuously hydrogen-bonded antiparallel β-barrel (**Figure 1**). The calyx of this lipocalin fold is open at one end allowing uptake and binding of several small lipophilic molecules. This was the reason why LCN2 was initially thought to have transport functions. However, there is increasing evidence that many lipocalins exhibit great functional diversity and take over specialized and pleiotropic activities. This is especially documented in the numerous functions that have been reported for LCN2. Kjeldsen and coworkers already demonstrated that human LCN2 can be secreted in three different forms, namely its 25-kDa monomer, a ~46-kDa homodimer, and a ~135-kDa heterodimer composed out of LCN2 and metalloproteinase-9 (MMP-9) (3). This prompted to the assumption that LCN2 might modulate neutrophil gelatinase activity (3). The murine counterpart was identified as a superinducible protein with a molecular weight of 24-kDa (SIP24) in growth arrested, murine Balb/c 3T3 fibroblasts that become massively induced in response to mitogens, fibroblast growth factor (FGF), epidermal growth factor (EGF), and serum (5, 6). Similarly, hepatic expression of mouse LCN2/24p3 dramatically increased during turpentine-induced acute phase response and the production was significantly stimulated by tumor necrosis factor-α (TNF-α) (7). These findings suggest LCN2/SIP24/24p3 as a key systemic reactant to local or systemic inflammatory disturbances that is modulated in its expression by many factors. In line with its function as an acute-phase protein, hepatocytes were identified as a major source of LCN2 production mediating hepatoprotective effects in acute liver injury and showing positive correlation to hepatic inflammation (8, 9).

Nowadays, there is additional ample evidence that LCN2 is a key molecule that regulates iron homeostasis. Although LCN2 itself does not have an intrinsic binding ability for iron, it can bind to siderophores, which are a group of diverse, small, high-affinity iron-chelating compounds that are secreted by microorganisms in response to iron limitation in their environment to increase iron uptake (10–12). Devireddy and coworkers proposed distinct iron-dependent activities for murine LCN2. They suggested that iron-laden LCN2 (holo-24p3) binds to a cell surface receptor termed 24p3R, is internalized, and releases its bound iron, thereby increasing the intracellular iron content (13). On the contrary, iron-free LCN2 (apo-24p3) internalized by binding to its receptor can associate with an intracellular iron chelating siderophore, take over bound iron and transfer it outside the cell, thereby reducing the overall intracellular iron concentration and inducing the BCL2-interacting protein BIM/BCL2L11 expression and stimulating cell apoptosis (13).

Studies in two independent generated gene-targeted *Lcn2*-deficient mice confirmed the essential iron-dependent role of LCN2 in the early stages of innate immune response (14, 15). Consistent with the function of LCN2 in regulating iron homeostasis, respective mouse models exhibited an increased sensitivity to bacterial infection under iron-limiting conditions (14, 15). It was suggested that neutrophils from *Lcn2* null mice were less able to inhibit bacterial growth compared with wild type neutrophils, confirming LCN2 as a bacteriostatic factor preventing bacterial siderophore-mediated iron acquisition (15). Comparative bacterial profiling of *Lcn2* null mice and wild type mice further demonstrated that the lack of LCN2 protein provokes expansion of siderophore-dependent bacterial species, resulting in significant changes in the intestinal microbiome composition and increased inflammatory activity in the gastrointestinal tract (16). Importantly, mice lacking *Lcn2* displayed a persistent colonization with segmented filamentous bacteria in the ileum that were associated with signs of autolysis in apical villi and detachment of epithelial cells from the basal membrane. Moreover, we have previously speculated that the expression of LCN2 is an intrinsic “help-me” sensor that, upon injury, develops a biological activity promoting production of neutrophil-attracting chemokines (17). Since neutrophils are regarded as the first line of defense in the innate arm of the immune system, loss of LCN2 would consequently prevent the effective clearance of respective microorganisms.

Interestingly, LCN2 also plays a physiological role in reproductive biology in both sexes. Originally termed as uterocalin, LCN2 is involved in the tissue remodelling processes in the uterus during pregnancy (18). Additionally, the expression of *Lcn2* varies according to hormonal fluctuations in the different estrous stages of mice (19, 20). Berger and coworkers reported that *Lcn2* null females have a significantly lower pregnancy rate compared with wild type animals (15). Investigating the underlying cause, the authors found that LCN2 is an essential component in modulating the membrane properties of the sperm in the fertilization process in male mice (21). Furthermore, in mouse models, infertility of both male and female animals was shown to be associated with increased expression of LCN2 in testis and ovaries (20, 22). However, to date, the molecular pathways of LCN2 and a possible interaction with its receptor in the reproductive tract are poorly understood.

LCN2 also has critical functions in the regulation of various aspects of energy metabolism and expenditure (23). LCN2 modulates expression of genes involved in β-oxidation in adipocytes, promotes β-cell function, and counteracts obesity-induced glucose intolerance suggesting LCN2 as an endogenous compensatory signal to counteract metabolic dysregulation in obesity through its anorexigenic activity (23, 24). In the central nervous system, LCN2 is a pronounced direct appetite suppressor and satiety signal in humans and mice modulating caloric intake as well as fat and lean mass content (25, 26). In mice, LCN2 impacts mitochondrial and peroxisomal function and integrity that directly impacts hepatic triglyceride balance, oxidative stress, and apoptosis (27). Finally, LCN2 was shown to be a key modulator of hepatic lipid homeostasis that *in vitro* and *in vivo* controls the formation of intracellular lipid droplets by regulating expression of Perilipin 5 that maintains the balance between lipogenesis and lipolysis (28). All these findings suggest that LCN2, besides its immunomodulatory functions, has strong impact on many aspects in shaping the energy metabolism.

**Figure 2:**
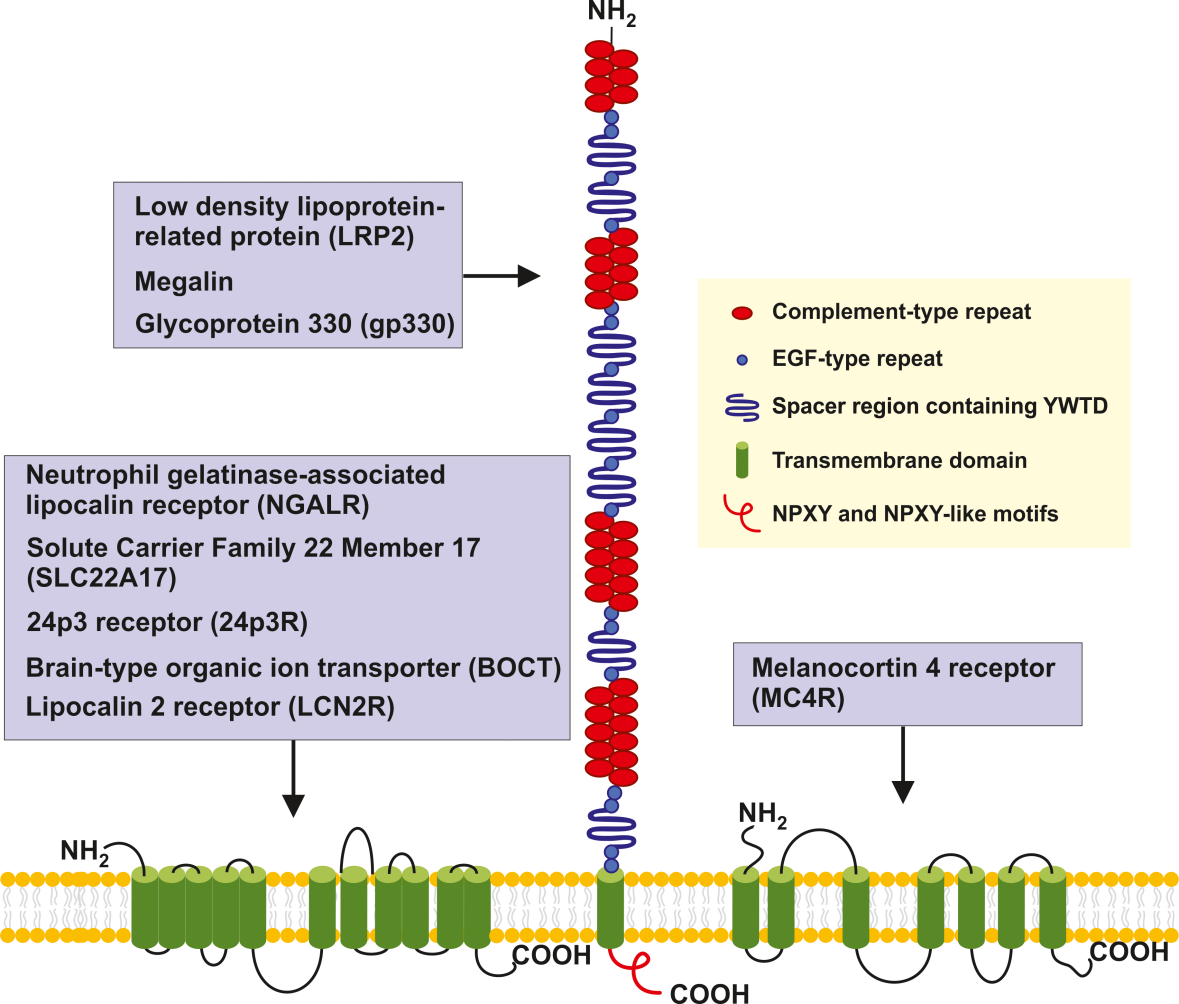
Structure and potential signaling pathways targeted by the putative LCN2 receptors. The three putative receptors for LCN2 are structurally different. NGALR also known as SLC22A17, 24p3, BOCT or LCN2R is a multipass 60-kDa integral membrane protein predicted to contain 11-12 transmembrane helices that are linked by extracellular and intercellular spacers of variable sizes (13, 29, 30). LRP2/megalin/gp330 is a ~4,600 amino-acid type 1 transmembrane receptor of the LDL receptor gene family characterized by extracellular domains containing four cysteine-rich clusters of complement-type repeats (i.e., the low-density lipoprotein-receptor type A repeats) that mediate ligand binding that are separated and followed by 17 epidermal growth factor type repeats and eight spacer regions that contain YWTD repeats. These are termed β-propellers that are required for pH-dependent release of bound ligands in endosomes (31). The single transmembrane of LRP2 encompassing 20 amino acids is followed by a 213 amino acid cytoplasmic tail, which contains two NPXY sequences and one NPXY-like sequence in addition to several Src-homology 3 (SH3) and one Src-homolog-2 (SH2) region sites (32). MC4R is a 332 amino acid G protein-coupled receptor (GPCR) with seven transmembrane helixes connected by alternating extracellular and intercellular loops. The recent structure of the human MC4R-G_S_ signaling complex bound to the agonist setmalanotide determined by electron microscopy demonstrated that the seven transmembrane-spanning helixes from a bundle that forms a cavity at the cytoplasmic side to accommodate the heterotrimeric G_s_ protein (33, 34). For simplicity the seven helixes are drawn as stand-alone transmembrane domains.

Unfortunately, all these insights into LCN2 function in innate immunity, iron homeostasis, and energy metabolism and expenditure are still descriptive. Precise mechanisms how LCN2 signals are transferred from outside into the cells are widely unknown and, although some biochemical activities and affinities were nicely demonstrated, there a numerous questions unresolved yet. In particular, the precise receptor activities and responsible pathways relevant in transmitting LCN2 inputs to intercellular targets are unknown. Actually, there are six putative LCN2 receptors, namely neutrophil gelatinase-associated lipocalin receptor (NGALR), low density lipoprotein-related protein 2 (LRP2), LRP6, melanocortin 4 receptor (MC4R), MC1R, and MC3R. The size and extent of their external ligand-binding domain, hydrophobic membrane-spanning regions, and their intracellular domains inside the cell are markedly different (**Figure 2**). Moreover, they are assigned to different chromosomes, reveal a marked different gene structure, and encode for mRNAs and proteins different in size (**Table 1**).

**Table 1:** Quick facts overview of putative LCN2 receptors in mouse and men*.

	NGALR	LRP2**	MC4R	MC1R	MC3R
**Alternate names**	SLC22A17 (H), 24p3R (M), BOCT (H), BOCT1/BOIT (H), LCN2R (H), NGALR2 (H), NGALR3 (H)	Megalin	Body mass index quantitative trait locus 20 (BMIQ20) (H)	melanocyte-stimulating hormone receptor (MSHR), melanotropin receptor (M, H)	MC3 (M, H), Body mass index quantitative trait locus 9 (BMIQ9) (H), HGMP01A (M)
**Classification**	Transmembrane transporter/signaling receptor	Multi-ligand scavenger receptor	G protein-coupled, seven-transmembrane receptors		
**Discovery**	Murine NGALR was identified as a receptor for LCN2 after three rounds of expression cloning in COS-7 cells using a cDNA library prepared from murine FL5.12 cells (13)	LRP2 was first identified in rats by screening a rat kidney λgt 11 cDNA expression library with antiserum raised against large, negatively charged antigens isolated from rat glomeruli. The initially isolated cDNA clone (C1B) was then used as a probe to isolate longer λgt 11 cDNA clones. Subsequently, a 15.4 kb cDNA encoding an open reading frame of 4660 amino acids was assembled by several rounds of screening of a random-primed rat kidney λZAP II cDNA library (35).	MCR4 was first isolated from a human genomic EMBL3 phage library that was probed with a fragment derived from a PCR amplified from mouse DNA using primers derived from conserved sequences located in the second intracytoplasmic loop and the seventh transmembrane domain of other members of the melanocortin receptor family (36).	Human and mouse MC1Rs were first isolated from melanoma cells (37, 38).	Human MC3R was first cloned from a human EMBL3 phage library using probes derived from PCRs conducted with primers located in the second intracytoplasmic loop and the seventh transmembrane region (39). Mouse MC3R was later isolated from a mouse genomic DNA library screened with a fragment derived from a PCR amplified with degenerative primers corresponding to consensus sequences of the third and sixth transmembrane segments (40).
Chromsosomal localization*	H: 14q11.2, M: 14C3	H: 2q31.1, M: 2C2	H: 18q21.32, M: 18E1	H: 16q24.3, M: 8E1	H: 20q13.2, M: 2H3
Gene structure*	10 exons	81 exons (H), 79 (M)	1 exon	1 exon	1 exon
mRNA transcript sizes*	H: 4 transcripts (2,426 nt, 2,372 nt, 2,037 nt, 2,037 nt) M: 5 transcripts (6362 nt, 6329 nt, 6225 nt, 2399 nt, 2294 nt)	H: 7 transcripts (15,657 nt, 15,591 nt, 15,815 nt, 15,531 nt 15,528 nt, 13,251 nt 13,248 nt) M: 15,429 nt	H: 1714 nt M: 2783 nt	H: 2,111 nt M: 3,670 nt	H: 1,084 nt, M: 2,623 nt
**Protein domains**	Signal peptide, major facilitator superfamily (MFS) domain (acting as single-polypeptide secondary carriers capable only of transporting small solutes in response to chemiosmotic ion gradients)	Signal peptide, 36 LDL-receptor class A (LDLRA) domain profiles, 35 LDL-receptor class (LDLRB) repeat profiles, 6 EGF-like domain profiles	G-protein coupled receptors family 1 signature with seven transmembrane regions		
Protein sizes*	H: 649 aa, 631 aa, 302 aa M: 520 aa, 401 aa	H: 4,655 aa, 4,612 aa, 4,347 aa, 3,892 aa M: 4660 aa,	H: 332 aa M: 332 aa	H: 317 aa M: 315 aa	H: 323 aa M: 323 aa
**Dissociation constant (*K*_d_) for LCN2 binding**	~ 92 pM (41) 7-10 µM (apo-LCN2 to NGALR aa1-aa105) and ~20 µM (LCN2/ferric-enterobactin to NGALRaa1-aa105) (30)	~ 60 nM (42)	51.39 ± 4.78 nM (43)	86.96 ± 9.72 nM (43)	82.13 ± 12.14 nM (43)
**Animal models**	Homozygote mutant mice are embryonic lethal [personal communication Yukio Nakamura, RIKEN BioResource Research Center].	Homozygote *Lrp2* mutant mice usually die perinatally from respiratory insufficiency within the first few minutes after birth or within 2-3 hours (44). However, there are also reports showing that *Lrp2* deficient mice can survive at low frequency (45).	Mice deficient for *Mc4r* develop a maturity-onset obesity syndrome associated with hyperphagia, hyperinsulinemia, and hyperglycemia (46).	The lack of *Mc1r* in mice results in a yellow coat in mice (47).	*Mcr3* deficient mice have increased fat mass, reduced lean mass and higher feed efficiency than wild type mice (48).

* Data was taken from different databases (OMIM, Gene, Genome) hosted by the National Library of Medicine (https://www.ncbi.nlm.nih.gov/). ** Recently, mouse LRP6 was shown to bind mouse LCN2 in co-immunoprecipitation assay (49). Abbreviations used are: aa, amino acids; H, human; M, mouse.

## 2 Neutrophil gelatinase-associated lipocalin receptor

*NGALR* (OMIM: 611461) is a gene that received a number of different names historically deduced from their proposed function or from its structure. The names NGALR and LCN2R were derived from the receptor’s affinity for LCN2. NGALR2 (or NgalR-2) and NGALR3 (or NgalR-3) refer to splice variants of NGALR that share overlapping biochemical characteristics (50). The name solute carrier family 22 member 17 (SLC22A17) indicates that the receptor belongs to the solute carrier (SLC) group of membrane transport proteins that commonly contain a number of hydrophobic transmembrane α-helixes. This family currently includes about 458 members that are classified into 65 families by similarities in their characteristics, homology, functions, and structures (51, 52). The SLC22 family contains about 30 distinct multi-membrane spanning proteins, 13 of which have been localized to plasma membrane (52, 53). Beside its affinity for LCN2, SLC22A17 binds to and mediates the endocytosis of filtered protein in the kidney and assists in iron uptake but does not drive the transport of other usual substrates of the SLC22 family (52). The names BOCT, BOCT1, and BOIT are abbreviations for brain-type organic ion transporter. The BOCT sequence (acc. no. NM_021551) popped up in the initial study reporting the expression cloning of murine NGALR (i.e., 24p3R) after searching the GenBank database with two identified overlapping NGALR sequences as queries (13). Finally, the introduced acronym 24p3R indicates that this receptor has affinity for murine LCN2 termed 24p3 (13, 41). 24p3R, which is now better referenced to as NGALR, was first identified as a LCN2 receptor after three rounds of expression cloning in COS-7 cells using a cDNA library prepared from murine FL5.12 cells, which are highly sensitive to LCN2/24p3-medicated apoptosis (13).

Homozygote *Ngalr* mutant mice are embryonic lethal (Yukio Nakamura, personal communication), suggesting that this receptor has already important activities in the developing embryo (54, 55). It is a transmembrane signaling receptor involved in insulin receptor recycling and transport of inorganic cations/anions, amino acids, and oligopeptides. Although NGALR has been shown to be involved in endocytosis, SLC22A17 is still considered as an orphan transporter for which no specific substrates or transport mechanisms have been identified (53). Nevertheless, potential functions of NGALR have been identified in various malignancies, including cancerogenesis, kidney disease, obesity, and many other diseases (53). In particular, it has been recently shown that the LCN2/24p3R axis is highly relevant in promoting nonalcoholic steatohepatitis in high-fat diet-fed Ob/Ob mice by activating hepatic stellate cells, representing the key pro-fibrogenic cell type within the liver (56).

Binding of mouse LCN2 to murine NGALR/24p3R overexpressed in human HeLa cells was first demonstrated by ligand-cell binding experiments, in which ^32^P-labeled 24p3 was bound to FL5.12 cells. Based on titration curves and Scatchard plot analysis, the dissociation constant of 24p3 to NGALR was determined to ~ 92 pM (41). Direct binding of apo-LCN2 to the *N*-terminal part containing the first 105 residues of human NGALR was further demonstrated by solution-state biomolecular nuclear magnetic resonance (NMR) in conjunction with other biophysical methods (30). However, in this study, the binding constant of both molecules as determined by isothermal titration calorimetry and microscale thermophoresis was determined to be in the low micromolar range (~7-10 µM), while the affinity of LCN2 complexed with ferric-enterobactin was even about threefold lower (30). This suggests that the *N*-terminus of NGALR exhibits some specificity toward apo-LCN2, while NGALR does not or only poorly bind to holo-LCN2 (30). Finally, it should be noted that the affinity determined by NMR-based techniques is about six orders of magnitude lower than that observed in the ligand-cell binding experiments. In addition, contradictory findings showed that exogenous LCN2 had no effect on iron release or uptake in NGALR-expressing HeLa cells and relevant NGALR subdomains failed to bind LCN2, questioning the overall assumption that NGALR and LCN2 form effective receptor-ligand complexes (29). This contributes to the general discussion about problems and current standards with reproducibility and rigor in conducting measuring and evaluating binding affinities with common high-throughput technologies (57). Additionally, the interaction of a ligand with its receptor in cell-ligand binding experiments is strongly dependent on the cell system used (58), suggesting that binding characteristics of ligands and potential receptors might differ between cells analyzed. Finally, biomolecule associations and affinities are further influenced by equilibration time, temperature, half-life of complex, salt concentrations, pH, amount of active (not total) protein, and many other factors (57).

## 3 Low density lipoprotein-related protein 2

LRP2 (OMIM: 600073) is a large multi-ligand, type-1 transmembrane receptor that was first identified in rats by screening a rat kidney λgt 11 cDNA expression library with antiserum raised against large, negatively charged antigens isolated from rat glomeruli (35). The initially isolated cDNA clone (C1B) was then used as a probe to isolated longer λgt 11 cDNA clones. Subsequently, a 15.4 kb cDNA was assembled by several rounds of screening of a random-primed rat kidney λZAP II cDNA library that contained an uninterrupted open reading frame encoding 4660 amino acids (35). LRP2 is an endocytic receptor sharing structural similarity with the LDL receptor, which in vertebrates is the main receptor for lipoprotein uptake. Ligands for LRP2 are plasma carriers transporting vitamins and steroid hormones, which upon binding, become internalized from the apical cell surface and sorted into different cellular pathways (31). LRP2 is structurally composed of a large extracellular domain consisting of (i) complement-type repeats that have affinity for different ligands, (ii) eight β-propeller folds built by six sheets composing four antiparallel β-strands and containing up to six Tyr-Trp-Thr-Asp (YWTD) or YWTD-like repeats, (iii) flanking epidermal growth factor (EGF)-like domains, (iv) a short single membrane-spanning region, followed by (v) a small C-terminus enriched in Asn-Pro-X-Tyr (NPXY) and NPXY-like motifs required for efficient ligand-mediated receptor internalization and biological signaling (59, 60).

As its alternative name megalin indicates, full length LRP2 with its 4665-4660 amino acids in mice and humans is a giant protein with a molecular weight of ~ 600 kDa. LRP2 can physically interact with the cellular communication network factor 2 formerly known as connective tissue growth factor (CCN2/CTGF), bone morphogenetic factor-4 (BMP-4), and sonic hedgehog signaling (SHH) molecule (60). Affinity of human iron-free LCN2 (apo-LCN2) and siderophore-bound LCN2 to human LRP2 was first demonstrated by surface plasmon resonance (SPR) analysis with matrix bound LRP2. In the respective experimental setup, the dissociation constant of human LCN2 to human LRP2 was determined to ~60 nM (42), which is quite similar to the *K*_d_ determined for the interaction of murine LCN2 and MC4R which was determined to 51.39 ± 4.78 nM (43).

It was supposed that megalin functionally blocks the passage of elevated plasma LCN2 occurring in sepsis, ischemia or nephrotoxic injury into the urine (61). In line, with this suggestion the deletion of megalin in proximal tubules of murine kidney resulted in proteinurea containing large quantities of LCN2 (61). Mutated forms, in which positively charged amino acids were mutated on the surface of LCN2 without disrupting neither the typical lipocalin fold nor the ligand-binding site of LCN2, lost their affinity for LRP2, showing that the ligand-receptor binding is majorly formed by electrostatic forces and the net positive charge displayed by LCN2 (61). LRP2 has also affinity for the retinol-binding protein (RBP), α_1_-microglublin (α_1_M), odorant-binding protein IA (OBPIA), major urinary protein 6 (MUP6), and apolipoprotein M (APOM) that all belong to the lipocalin family (62, 63). Based on these findings, it might be possible that individual domains within LRP2 act as unspecific scavengers that recognize a broad range of ligands by their three-dimensional fold such as the symmetrical β-barrel fold that is a unifying characteristic of lipocalins. Nevertheless, the binding of other lipocalins with *K*_d_ values in the µM range such as RBP (*K*_d_ = 1.8 µM) and α_1_M (*K*_d_ = 0.42 µM) to megalin have significantly lower affinities than that of LCN2 (*K*_d_ = 60 nM) (42, 63, 64). Moreover, the affinity of purified human or rat LRP2 to other proteins such as the lipoprotein lipase is 10 to 30 fold lower (*K*_d_ = 6.1 and 2.7 nM, respectively) than that of LCN2 (65). Nevertheless, since LRP2 is involved in the endocytosis of a wide range of molecules (LCN2, siderophore-bound iron, diverse lipoproteins, hemoglobin, albumin, hormones, toxins, drugs, vitamin-binding proteins, stress-related proteins and many others), LCN2 when overexpressed during malignancies might change the affinity for other LRP2 binding partners by blocking respective binding sides (66).

Recently, mouse LRP6 composed of the same basic structural motifs as LRP2 acting as a co-receptor for Wnt was shown to specifically bind to mouse LCN2 (49). In the respective study, the interaction of LCN2 and LRP6 was shown in co-immunoprecipitation assay and it was suggested that binding of LCN2 to LRP6 effectively inhibits Wnt/β-catenin signaling. Interestingly, LRP5 which is about 70% homologous to LRP6 and also active as a co-receptor in Wnt signaling failed to bind LCN2 (49, 67). However, information about the strength of the binding of LCN2 and LRP6 and independent confirmation of observed interaction in other species than mouse is still pending.

## 4 Melanocortin receptors

MC4R (OMIM: 155541) belongs to a family of G protein-coupled 7-transmembrane receptors that contains five family members (termed MC1R to MC5R). The individual members of that family share a sequence homology of 38 to 60% (68). Each receptor has different specificity for melanocortins that consists of adrenocorticotropin hormone (ACTH), α-melanocyte-stimulating hormone (α-MSH), β-MSH, and γ-MSH (68). Receptor activation results in formation of cyclic AMP (cAMP) and activation of protein kinase C (PKC) that in turn provokes influx of extracellular calcium resulting in inositol triphosphate (IP3) production and downstream activation of MAPK and JAK-STAT pathways (68).

In regard to LCN2, it was further demonstrated that osteoblast-derived LCN2 can cross the blood-brain-barrier and bind to MCR4 in paraventricular nucleus neurons of the hypothalamus as assessed by application of biotinylated Lcn2 in *Lcn2* null mice (43). In addition, the overexpression of MC4R in HEK293T cells dose-dependently stimulated cAMP activity with an EC_50_ of 1.41 ± 0.25 nM, while silencing or pharmacological inhibition of MC4R with the MC3R/MC4R antagonist SHU9119 abrogated Lcn2-activated cAMP production and signaling in the mouse hypothalamic, gonadotrophin-releasing hormone neuronal cell line GT1-7. This inhibition was independent from the two other putative Lcn2 receptors (i.e. MC1R and MC3R) because LRP2 is not expressed in GT1-7 cells and silencing of NGALR did not affected LCN2 signaling (43). Saturation and competition binding experiments in MCR4 transfected HEK293T showed that LCN2 binds to MC4R with a dissociation constant *K*_d_ of 51.39 ± 4.78 nM. This affinity is about 4.5-fold lower than that of the classical MCR ligand α-MSH.

In the same study, the authors showed that LCN2 also has affinity for MC1R (*K*_d_ = 86.96 ± 9.72 nM) and MCR3 (*K*_d_ = 82.13 ± 12.14 nM) and can stimulate cAMP production through these receptors. However, the authors suggested that MC4R is the most relevant MCR for LCN2 signaling in paraventricular and ventromedial neurons because MC1R and MC3R are not able to regulate appetite (43). Similar to MC4R, MC3R is a neural MCR that is expressed primarily in the central nervous system having important functions in regulating feed efficiencies by binding to identical ligands with similar binding affinities (69). The third putative MC receptor for LCN2, MC1R, is expressed in neurons, astrocytes, and microglia and also sensitive to the endogenous nonselective agonist α-MSH (70). The activation of MC1R can prevent neutrophil infiltration, thereby reducing the overall LCN2 content that is available within the brain (71). Furthermore, because LCN2 participates in the pathogenesis of blood-brain barrier disruption, MC1R with its lower affinity might be a kind of LCN2 sensor capable to prevent overshooting LCN2 concentrations within the brain by blocking neutrophil infiltration (72).

In addition to its activity in control of appetite, MC4R can directly impact glucose and lipid homeostasis, pain perception and even sexual behaviors by modulating of luteinizing hormone and prolactin surges in female and erectile activity in male rodents (73, 74). However, if these MC4R-driven activities can be modulated by LCN2 is not known yet.

## 5 Signaling pathways for putative LCN2 receptors

**Figure 3:**
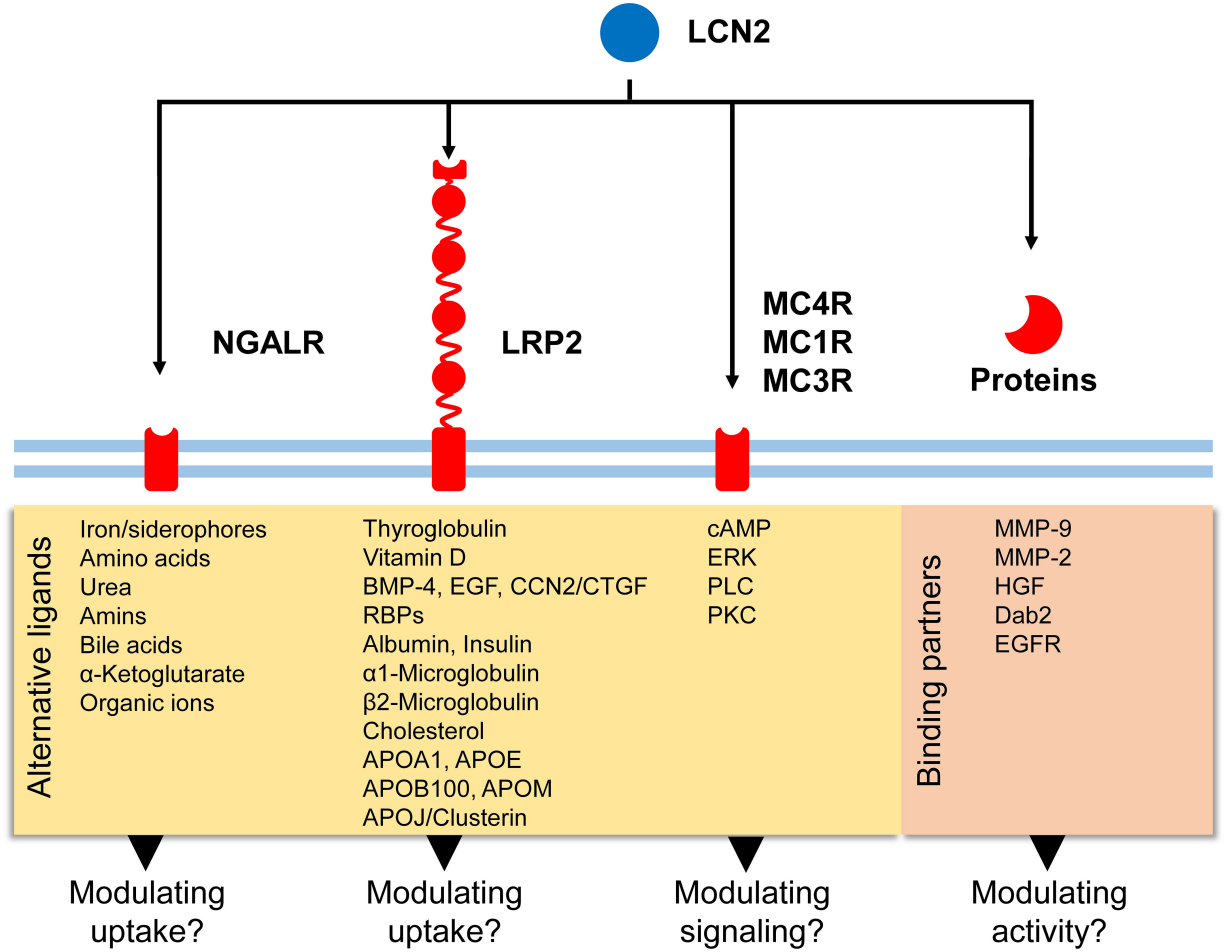
Potential signaling routes for LCN2. LCN2 has affinity for five putative receptors (NGALR, LRP2, MC4R, MC1R, and MC3R) and additional binding partners (*e.g.*, MMP-9, MMP-2, HGF). Binding of LCN2 to these biomolecules can modulate activity of respective proteins/receptors or modulate signaling cascades triggered. In addition to these receptors, LCN2 was recently found to bind to LRP6 in mouse embryonic fibroblasts (49).

As discussed, there are six putative receptors supposed to have (more or less affinity) for LCN2. The receptors either belong to the group of transmembrane transporter/signaling receptors (NGALR), multi-ligand receptors (LRP2, LRP6), or G-protein coupled seven transmembrane receptors (MC4R, MC1R, MC3R). If these putative receptors are indeed *bona fide* LCN2 receptors, LCN2 would have many possibilities to modulate cellular signaling *via* binding to one of these receptors or by binding combinations of these receptors (**Figure 3**).

NGALR belongs to the SLC superfamily of proteins that can transport a wide array of molecules, including sugars, amino acids, oligopeptides, vitamins, nucleotides, metals, inorganic ions, organic anions, and drugs (52). Within the SLC group, NGALR belongs to the major facilitator superfamily (MFS) group that in particular transport organic ions including urea, amines, bile acids, and α-ketoglutarate (52). Importantly, NGALR assists in the uptake of iron and shows a more restricted substrate profile than other SLC members, suggesting that NGALR has more specialized physiological functions (75). It was suggested that NGALR contributes to adaptive osmotolerance by controlling the uptake of amino acids as osmolytes and that LCN2 downregulation counteracts increased proliferation and permanent damage of osmotically stressed cells (76). Similarly, it was proposed that murine LCN2 alters cellular iron trafficking through iron efflux *via* murine NGALR, thereby driving apoptosis (41). In line with this assumption, exogenous LCN2 sensitized primary cortical neuronal cells and neuroblastoma cell lines to cell death (77). Likewise, the addition of LCN2 increased intracellular reactive oxygen species formation, DNA damage, cell cycle arrest and cell death in mouse fibroblast cell line L929 (78). Additionally, in both acute and chronic white matter diseases in the brain, it could be shown that LCN2 has an inhibitory effect on the oligodendrocyte differentiation necessary for remyelination, which was mediated *via* SCL22A17/EGR1 signaling (79).

We showed that the lack of LCN2 is associated with endoplasmic stress, unfolded protein response, and modulation of the cellular labile chelatable iron pool (27, 80). However, there are also findings showing that LCN2 has protective and survival activity (9, 81), while other studies have shown that exogenously added LCN2 in HeLa cells that overexpress NGALR did not affect iron efflux or uptake (29). Similarly, there are numerous other studies that show conflicting data in the understanding of LCN2’ function in controlling apoptosis.

LRP2 is a multi-ligand binding receptor that has important roles during embryogenesis by supplying the fetus with nutrients and controlling sonic hedgehog signaling (31). It further serves as a receptor for thyroglobulin (82) and Vitamin D (83), has affinity for BMP-4 (83), forms specific complexes with cubulin and Dab2 that mediate the uptake of filtered proteins (e.g., vitamin-binding proteins, retinol-binding proteins, albumin, insulin, α_1_-microglobulin, β_2_-microglobulin, EGF, and lysozyme) and escape the glomerular filtration barrier and driving the endocytic uptake of clathrin-coated vesicles (84). It further has affinity for several ligands involved in cholesterol metabolism (e.g., APOA1, APOE, APOB100, APOJ/clusterin, and APOM) and is important for the endocytosis of high density lipoprotein particles (83). So there is large variety of activities that can be modulated by interacting of LCN2 with specific modules within LRP2.

As discussed, the MCRs (i.e., MC4R, MC1R, and MC3R) supposed to have affinity for LCN2 are belonging to the group of 7-transmembrane G-protein linked receptors. From these receptors MC4R has the highest affinity for LCN2. Its activation leads to increased intracellular cAMP (36, 39). Moreover, more recent studies have shown that MC4R is able to activate the extracellular regulated kinase (ERK) and modulate the activity of other G proteins receptors, most likely by sharing affinity for the same G-proteins that mediate signaling *via* these receptors (85, 86). This is in line with previous findings showing that the G protein G_s_α is required for controlling MC4R-regulated energy expenditure and glucose metabolisms, while effects on food intake, linear growth, cholesterol metabolism, and expression of specific transcription factors in the paraventricular nucleus of the hypothalamus (i.e., *Sim1*, *Crh*) are primarily mediated by the G_q/11_α subunit (87, 88). In addition to G_s_α and G_q/11_α, MC4R can also couple to other G proteins such as Gαi, which inhibits the adenylate cyclase activity, thereby decreasing intracellular cAMP levels, and Gαq, which activates phospholipase C (PLC) and downstream PKC (89). Presently, it is unknown if LCN2 can modulate the affinity of MC4R for G proteins. Nevertheless, LCN2 was shown to increase the formation of cAMP as efficiently as 0.5 nM α-MSH in mouse GT1-7 hypothalamic cells, but failed to activate phosphorylation of AMPK, ERK1, ERK2, or tyrosine kinase in these cells, further suggesting that cAMP formation and kinase phosphorylation might not be necessarily linked to each other (43).

So in sum, LCN2 seems to have many possibilities to interfere with cellular signaling through its putative receptors. This notion is underpinned by the finding that the overexpression of LCN2 in the esophageal squamous cell carcinoma cell line EC109 resulted in the upregulation of 167 genes and downregulation of 96 genes using a 2-fold threshold encoding genes that have further capacity to interact with thousands of other proteins (90). This study that was based on protein-protein interaction network showed that LCN2 is a highly pleiotropic protein having affinity for many other proteins, thereby causing a wide range of expression alterations in immunity-related terms, pathway-related terms, cellular response to molecules of bacterial origin, extracellular matrix organization, and cell cycle-related terms. However, there is an urgent need to clarify if these effects of LCN2 are mediated by acting as a signal-inducing ligand at a specific receptor, masking binding epitopes for other proteins on proposed LCN2 receptors, binding to other biomolecules (e.g., proteins, hormones, vitamins etc.) preventing their interaction with other proteins/receptors, or alternatively by directly or indirectly changing general cellular features (osmolarity, iron content, mitochondrial activity). It should be noted that the outcome of the induced biological effect is the sum of the different interaction levels with all receptors expressed at a specific time in a specific environment and the accessibility of possible target structures at the time of LCN2’ presence. This should results in an extreme high combinatorial diversity of LCN2 activities.

## 6 Unsolved issues in LCN2 receptor signaling

### 6.1 Experimental aspects

**Figure 4:**
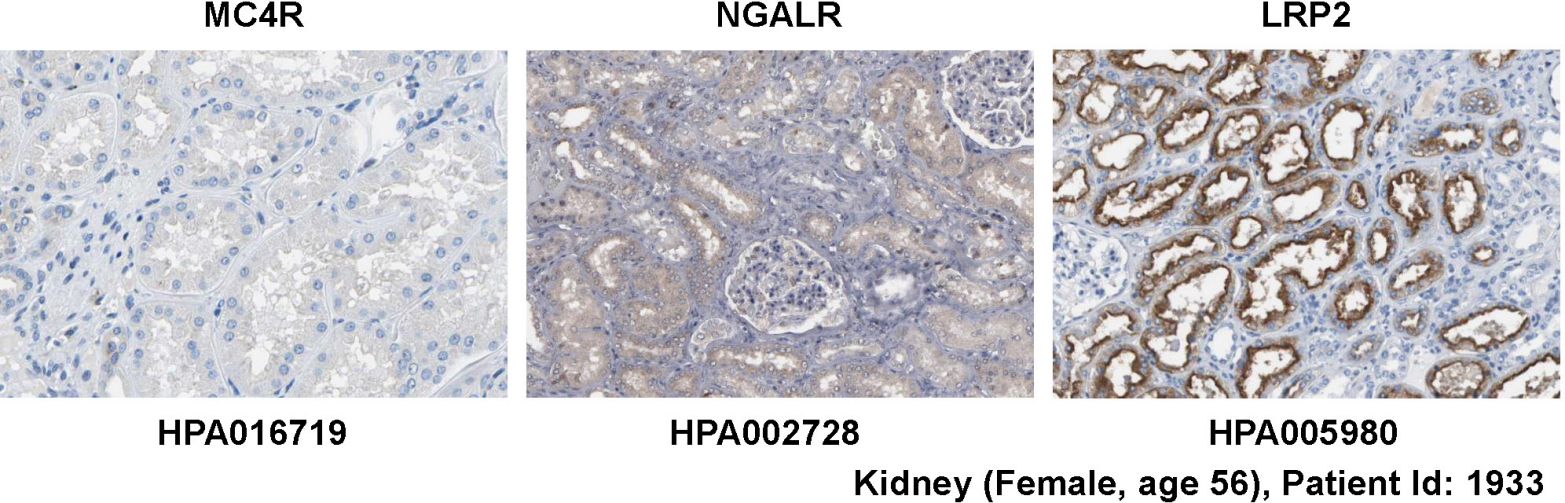
Expression of potential LCN2 receptors in kidney. Normal kidney tissue sections were stained with antibodies specific for MC4R, NGALR, and LRP2 showing that the kidney lacks MC4R expression. All images were taken from the Human Protein Atlas (91) database [https://www.proteinatlas.org/ 92]. The images can be found at: https://www.proteinatlas.org/ENSG00000166603-MC4R/tissue/kidney#img (*left*), https://www.proteinatlas.org/ENSG00000092096-SLC22A17/tissue/kidney#img (*middle*), and https://www.proteinatlas.org/ENSG00000081479-LRP2/tissue/kidney#img (*right*).

**Figure 5:**
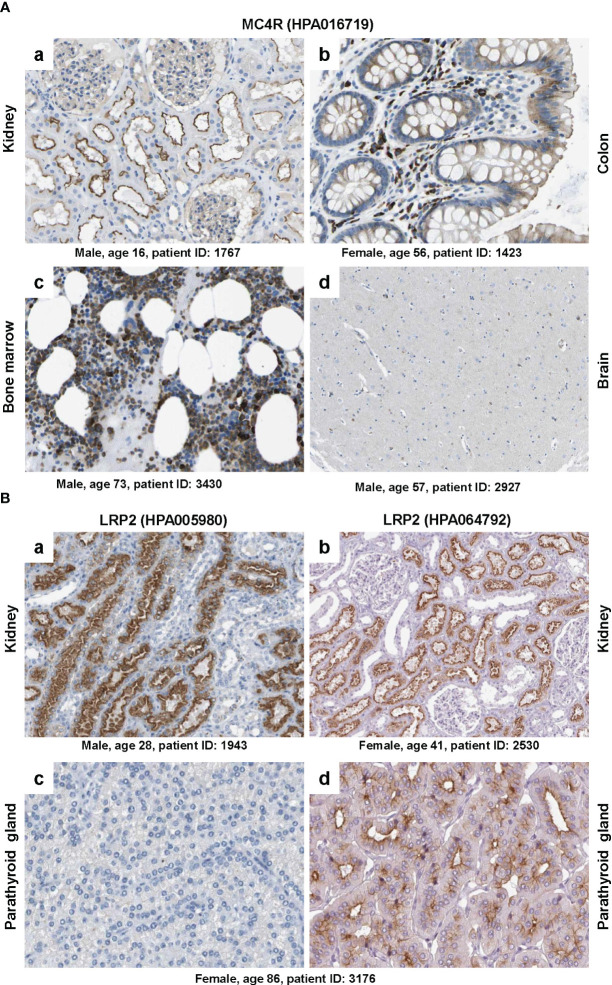
Immunohistochemical staining of MC4R and LRP2/megalin. **(A)** Tissue sections from (a) kidney, (b) colon, (c) bone marrow and (d) brain were stained with a validated antibody (HPA016719) specific for MC4R. Please note that the antibody stained tubular cells in the kidney, hematopoietic cells in the bone marrow, glandular cells in the colon, while only showing low staining in some neuronal cells in the brain. **(B)** Tissue sections from (a, b) kidney and (c, d) parathyroid gland were stained with two (validated) polyclonal rabbit antibodies (i.e., HPA005980 and HPA064792) directed against human LRP2. Please note that both antibodies stained similar structures in kidney, while the staining was markedly different in two sections of the parathyroid gland that were taken from the same patient. All images were taken from the Human Protein Atlas (91) database [https://www.proteinatlas.org/ 92]. The images depicted in **(A)** can be found at: (a) https://www.proteinatlas.org/ENSG00000166603-MC4R/tissue/kidney#img, (b) https://www.proteinatlas.org/ENSG00000166603-MC4R/tissue/colon#img, (c), https://www.proteinatlas.org/ENSG00000166603-MC4R/tissue/bone+marrow#img, and (d) https://www.proteinatlas.org/ENSG00000166603-MC4R/tissue/cerebral+cortex#img. The images depicted in **(B)** can be found at: (a, b) https://www.proteinatlas.org/ENSG00000081479-LRP2/tissue/kidney#img and (c, d) https://www.proteinatlas.org/ENSG00000081479-LRP2/tissue/parathyroid+gland#img.

NGALR is expressed in many organs and tissues (13), while the expression of the neuronal MC4R receptor as well as MC1R and MC3R are more restricted and found primarily in the brain (36, 37, 39). The expression of LRP2 is high in kidney, less in lung, and low in liver, brain, heart, and skeletal muscle (35). In line with the proposed expression patterns, MC4R protein is not detectable in kidney, while NGALR protein is detectable and LRP2 protein is found in high abundance within the kidney (**Figure 4**). Nevertheless, based on our own experience and discussion with many colleagues, it should be mentioned that the uncritical usage of antibodies directed against the putative LCN2 receptors can lead to significant errors. Some examples of discrepant and conflicting experimental results for staining with putative LCN2 receptor antibodies are given in **Figure 5**, depicting data taken from the open access Human Protein Atlas resource, which uses only “validated antibodies” that have undergone a strict quality control process with defined criteria (Human Protein Atlas).

#### 6.1.1 Example 1: Cross-reactivity of antibodies directed against specific LCN2 receptors

The polyclonal rabbit affinity isolated antibody HPA016719 directed against a recombinant human MC4R peptide produced strong signals on sections taken from kidney, colon and bone marrow, while only some neural cells in the brain were positively stained for MC4R (**Figure 5A**). However, MC4R as a neuronal receptor should be expressed abundantly in caudal brainstem structures (73) but not in kidney, colon or bone marrow. Because HPA016719 was produced against a 44 amino acid protein epitope signature tag (PrEST) located at the *N*-terminal part of human MC4R (aa-9-aa52; NH_2_-MVNS……YEQL-COOH), one could mistakenly conclude from the observed staining pattern that parts of MC4R containing the *N*-terminal region of MC4R are released from the brain and taken up or bound by cells located in the kidney, colon, and bone marrow that do not themselves express MC4R. But how likely is that?

#### 6.1.2 Example 2: Failure of an antibody to bind to its antigen

Another striking example is depicted in **Figure 5B** showing that staining with two polyclonal rabbit affinity isolated antibodies (HPA005980 and HPA064792), both directed against human LRP2, resulted in highly consistent results in kidney, while the parathyroid gland, known to express large quantities of LRP2 (93, 94), was only significant stained with antibody HPA064792. This finding is extremely surprising because the two antibodies were tested on sections taken from the same patient (patient ID: 3176). Of course this discrepancy might result from the fact that both antibodies were prepared against different recombinant protein fragments of human LRP2. Antibody HPA005980 was produced against PrEST consisting of aa1333 to aa1482 of human LRP2 (NH_2_-GFT…….STDL-COOH) and antibody HPA064792 against a PrEST spanning aa4240 to aa4331 (NH_2_-NND……….SVP-COOH) of human LRP2. Therefore, one could argue that the parathyroid gland expresses a shortened, *N*-truncated LRP2 isoform that is not recognized by antibody HP005980. But again, how likely is that assumption?

These examples are highly alarming and there are many other examples showing that uncritical usage of antibodies directed against LCN2 receptors might be a source for faulty data. Unfortunately, companies selling these antibodies propose “*The antibodies that have been generated … have been tested by immunohistochemistry against hundreds of normal and disease tissues*” and “*The uniqueness and low cross-reactivity of the antibodies to other proteins are due to a thorough selection of antigen regions, affinity purification, and stringent selection*”. It is obvious that such statements give false certainty about the quality of respective antibodies.

Moreover, there are many antibodies available for NGALR, LRP2 and MC4R that have not undergone strict quality controls. Some of them are even advertised to be specific for several species and applicable for immunohistochemistry, Western blot analysis, flow cytometry, and ELISA testing. Nevertheless, the ones that we have tested in our laboratories even failed to recognize high quantities of transiently expressed proteins in cell extracts or produced unspecific, un-interpretable background signals.

### 6.2 Functional aspects

There are also many functional aspects in LCN2 receptor biology that are controversially discussed or unexplained. In the following, two examples are briefly discussed to clarify what is meant.

#### 6.2.1 Example 1: Specificity of LCN2 activities

**Figure 6:**
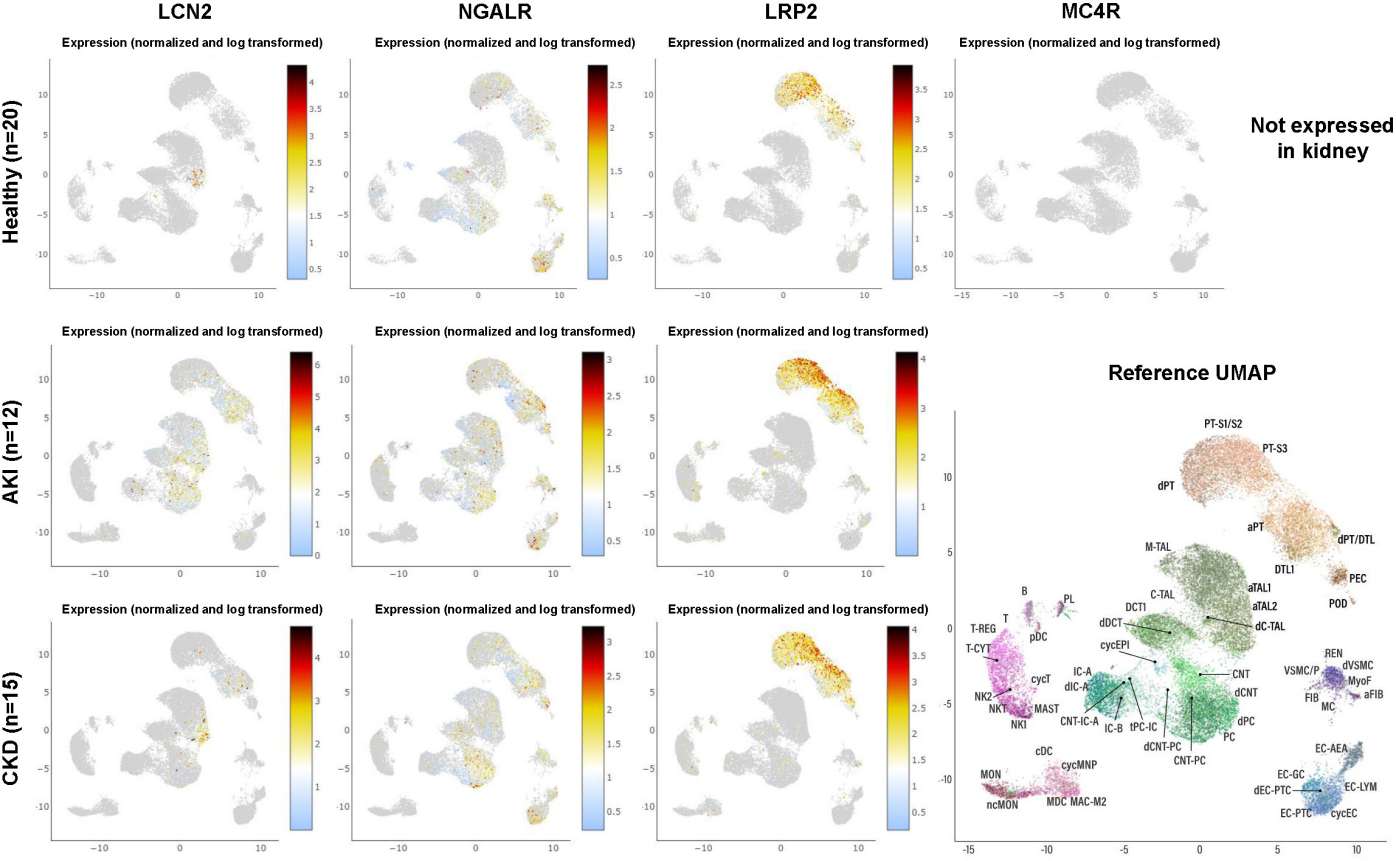
Expression of LCN2 and its putative receptors in human kidney in health and disease as obtained from single-cell RNA-sequencing. The dataset comprises 20 samples from 18 living donor biopsy participants taken from the Human Cell Atlas, as well as 15 biopsies from patients suffering from chronic kidney disease and 12 biopsies taken from patients suffering from acute kidney injury. For individual abbreviations depicted in the two-dimensional Reference Uniform Manifold Approximation and Projection (UMAP) image, please refer to **Supplementary Table 1**. Please note that the cell subsets that are capable to express LCN2 increase during renal disease, while the expression profile of the putative LCN2 receptors NGALR and LRP2 does not change. Moreover, renal expression of LRP2 is more restricted and MC4R is not expressed at all in any kidney cell fraction. The results here are in whole or part based upon data generated by KPMP [https://atlas.kpmp.org]. The respective URL address used for visualizing expression data from LCN2, NGALR, LRP2, and MC4R in healthy kidney, acute kidney injury and chronic kidney disease is: https://atlas.kpmp.org/explorer/dataviz. All data were downloaded on 20 May, 2023.

Numerous studies have shown that the expression of LCN2 is a crucial sensor that indicates inflammation, cell damage, stress and many other factors. As such, LCN2 is an important factor contributing to the cellular microenvironment and has pivotal vial roles in cell-cell communication. Nevertheless, there are many different cell types and organs that can produce and secrete elevated quantities of LCN2 during injury into the systemic circulation. Consequently, it is questionable how overexpressed LCN2 can mediate specific effects in a specific organ or tissue. Exemplarily, the expression of LCN2 is significantly increased in several renal cell types during acute and chronic kidney injury (**Figure 6**). Consequently, this causes increased LCN2 quantities in the blood. Based on the fact that LCN2 can pass the blood-brain barrier (25), kidney insult should be accompanied with increased cerebral quantities of LCN2 and subsequent activation of MC4R, thereby modulating food intake and energy expenditure. However, it is already known for decades that the energy expenditure in patients with renal failure is not different from normal (95). Similarly, patients suffering from diverse liver insults have increased concentrations of LCN2 (17). Consequently, elevated concentrations of LCN2 occurring during fatty liver disease induced by elevated energy intake should limit progression of hepatic disease itself through MC4R-dependent suppression of appetite. However, quite the opposite is the case.

#### 6.2.2 Example 2: Function of LCN2 in the pathogenesis of insulin resistance

In mice, the disruption of *Lcn2* resulted in insulin resistance, which significantly potentiated diet-induced obesity and fatty liver disease (96). However, this finding is in contrast to other studies showing (i) that LCN2 deficiency in mice protects against developing aging- and obesity-induced insulin resistance (97), (ii) elevated quantities LCN2 contribute to insulin resistance in humans (98), and (iii) increased LCN2 expression acts as a protective mechanism to counteract obesity-induced glucose intolerance by decreasing food intake and promoting adaptive β-cell proliferation (23). These controversial findings are hardly to explain. It becomes even more complicated if the observed effects should be mediated by specific receptors. LRP2, for example, acts as an endocytic receptor for reabsorption of insulin (99) and shows increased expression in early type 2 diabetes (100). Contrarily, MC4R deficiency in humans and rodents induce insulin resistance and obesity but do not develop hyperglycaemia (101), which is in line with the finding that MC4R agonists improve insulin secretion in diabetic mice (102). However, since NGALR is supposed to have the highest affinity for LCN2 from all putative receptors, this gap of knowledge is incomprehensible. In sum, it is obvious that additional work is required to understand LCN2’s roles in the pathogenesis/prevention of insulin resistance.

### 6.3 Lack or wrong information about antibodies

Additional confusion results when authors miss to provide detailed product information about the antibodies used in their study. In order to be able to reproduce a published experiments, it is of fundamental importance to have information about the full antibody name, clone number (if monoclonal), manufacturer, catalogue number, lot number, working dilution, detected epitope, and strategies or references demonstrating antibody validation. Unfortunately, only a small percentage of papers in the LCN2 receptor field provide respective information. Even more, missing proper controls, nonspecific binding, or using wrong antibodies for a specific antigen provoke erroneous and inconsistent results. Two examples taken from the LCN2 receptor field should be briefly discussed.

#### 6.3.1 Example 1

In a study from year 2020, Chen et al. (103) investigated the association of hepatic and systemic LCN2 levels in liver fibrosis, portal hypertension, and disease severity in patients with alcoholic hepatitis and in mouse subjected to ethanol or carbon tetrachloride treatments (103). In agreement with many previous studies, the authors found that LCN2 serum levels and gene expression correlated well with disease severity, liver fibrosis, and portal hypertension. Moreover, when comparing wild type and *Lcn2* null mice, the authors of that study showed that mice lacking *Lcn2* developed less fibrosis when subjected to carbon tetrachloride. They further showed an immunofluorescence staining for NGALR in liver specimen from patients suffering from alcohol hepatitis showing a strong upregulation of NGALR compared to healthy controls. When looking for the identity and source of antibody used in this study, the authors provide information that the NGALR antibody was from Sigma-Aldrich (#HPA049718). However, looking at the datasheet of mentioned antibody, the antibody is produced in rabbit and directed against the atypical chemokine receptor 3 (ACKR3), also known as CXC motif receptor 7 (CXCR7) or G protein-coupled receptor 159 (GPR159). Unfortunately, the respective antibody used in this study has nothing to do with NGALR, questioning the finding that NGALR is increased expression in human livers during alcohol induced liver damage.

#### 6.3.2 Example 2

Du and colleagues reported effects of LCN2 on brain endothelial adhesion and permeability (104). In their study, they used human brain microvascular endothelial cells (HBMEC) and showed an immunohistochemistry for NGALR and LRP2. Based on the results, the authors stated that the results confirmed that both receptors are expressed in HBMEC. Unfortunately, the authors show no proper controls, they only stated in the Material and methods sections “*Negative controls were incubated without primary antibodies and no immunoreactivity was observed in these controls*”. In our view, the chosen control is insufficient. A typical falsity in immunohistochemistry is the use of negative controls in which the primary antibody is omitted. Results of such studies show only the potential non-specific binding of the secondary antibody on the tissue, but not that of the primary antibody. The use of serum or isotype-specific immunoglobulins in the same protein concentration as the primary antibody is suitable as a negative control for staining (105).

In addition, the chosen primary antibodies used for NGALR or LRP2 might recognize other proteins. In regard to the source of antibody the authors provide the information that the LRP2 antibody was obtained from Abcam (Cambridge, MA) and the antibody against 24p3R was obtained from Bioss (Woburn, MA). Abcam presently offers seven different unconjugated antibodies against LRP2 (#ab76969, #ab309086, #ab309087, #ab236244, #ab223754, #ab101011, and #ab56014) from which six are recommend for immunohistochemistry in human materials. Bioss sells only one unconjugated polyclonal antibody against 24p3R/SLC22A17 (bs-044R) with reactivity against human NGALR (and rat). So it is obvious that the authors of respective study used this NGALR antibody for staining, which is also likely because the paper is listed on the companies’ homepage as one reference for this antibody. But which antibody was used for LRP2 staining and how was the specificity of both antibodies tested in the respective study?

In our laboratories, we have tested several antibodies against LRP2 and NGALR. Unfortunately, most of these antibodies showed unspecific background signals in cells and tissues. For example, a rabbit polyclonal LRP2 antibody (Elabscience, #E-AB-63748) showed a nuclear and cytoplasmic staining pattern in murine brain-derived endothelial cell line bEnd3, while a typical membranous staining supposed for a receptor was missing (not shown). Therefore, we would never argue that the staining is specific for LRP2 because some nuclei stained positive and more important bEnd3 are known to express large quantities of LRP1 (106), which shares an overall sequence identity of 38.40% with murine LRP2 as determined by blastp suite-2 alignment between respective protein sequences (i.e., sequence CLUSTW (mLRP1: EDL24513.1, mLRP2: NP_001074557.1). In addition, this antibody was produced against a recombinant fusion protein of human LRP2 for which the company does not provide further information (e.g., precise epitope) in respective datasheet. Based on the occurrence of individual modules (e.g., complement-type repeats, eight β-propeller folds, EGF-like domains, NPXY motifs) within LRP2 that are also found in similar structure and arrangement in many other proteins, it would be scientifically unaccountable to claim that the stain is specific for LRP2.

Similarly, staining with a polyclonal rabbit antibody directed against a peptide located in the C-terminal region of human NGALR (Invitrogen, #PA5-103667) produced in our hands only a faint stain in murine kidney and liver tissue that was only marginal stronger than the staining with an unspecific IgG control (not shown). NGALR in mouse kidneys should be highly expressed in apical membranes of distinct distal tubular segments (i.e., the distal convoluted tubule) in renal cortex and medulla as demonstrated already one decade ago by staining of tissues with a highly specific antibody (α-CT-24p3R) directed against the peptide GALPPNASGWEQPPNSC (the first 16 amino acids of that peptide correspond to amino acid 18 to 33 of murine NGALR) (107). Of course, we could argue that distal convoluted tubules are stained over background, but based on the overall low signal is this really scientifically robust? On the contrary, NGALR expression with this antibody was detected within the seminiferous tubules in murine testis. This expression seems to be specific because we could confirm the expression of NGALR by RT-PCR and subsequent sequencing of the resulting amplicon (not shown). This would be comparable to the staining results in rat testis, where NGALR was observed in the germ cells (localized in the seminiferous tubules) (108). Unfortunately, it was not further specified which antibody (from abcam) was used. However, usage of another NGALR antibody (LS-C53320, LS Bio) that was produced using a 14 amino acid synthetic peptide taken from the last 50 amino acids from the C-terminus of human NGALR and proposed to be cross-reactive for murine NGALR (Comment by the company: *test or 100% immunogen sequence identity*) failed to recognize murine NGALR with the expected size of ~ 58 kDa in Western blot analysis, while showing endogenous NGALR in Hep3B and HEK293 cells and transient overexpressed NGALR (not shown). However, in addition to this band, there is one (in HEK293) or two (in Hep3B) other protein band detectable in the higher molecular size range. So, could these bands be ignored, are these artifacts arising from inadequate protein reduction, or are these cross-reactive band to other sequence related proteins?

Moreover, when performing direct immunofluorescence analysis with another rabbit polyclonal Alexa Fluor^®^ 488-labeled antibody directed against a KLH-conjugated synthetic peptide derived from human SLC22A17 that should be cross-reactive to rat and mouse NGLAR (Bioss, #bs-0444R-A488), all weak signals for NGALR were found in kidney, liver, and testis (not shown). So what is specific and what not?

In sum, there are many experimental findings that are not easily to explain. In our laboratory, we have started to critically evaluate LCN2 receptor antibodies. We will be happy to discuss and share our experience on that topic upon reasonable request.

### 6.4 From mouse to men?

Work in mice has provided tremendous insight into the biology of LCN2. In particular, as research tools, mice studies have shown that *Lcn2* expression is significantly increased in many organs during stressful conditions and inflammation. Moreover, the finding that LCN2 binds siderophores has unraveled LCN2 as a critical component of the innate immune systems. Similarly, the identification of different receptors that have affinity to LCN2 has shield light on potential pathways by which LCN2 transmits signals into the cells. However, it is worth considering the possibility that findings obtained in mice may not precisely occur in the same way as in humans (109). In particular, there has been an intensive discussion in the past decade if mouse models mimic human inflammatory diseases (110, 111). Systematic studies investigating the animal-to-human translational success rates have shown that the translational success is unpredictable (112). Therefore, the question arises how valuable are all these mouse findings for humans and which findings can be translated to the pathophysiology of human diseases? In this regard, the low percent identity of human and mouse LCN2 that is only 62% has to be considered (**Table 2**). Compared to their putative receptors sharing up to 94% sequence homology, the two LCN2 orthologs in mouse and men are rather different. Despite the common assumption that orthologs usually share the same biological functions, there have been several reports of divergence between orthologs from mice and humans (114). There are some important examples showing that LCN2 and LCN2/p24p3 as one-to-one orthologs might have dissimilar functions in regard to their biochemistry and function. Two of these examples should be briefly discussed in the following.

**Table 2:** Sequence identity of murine and human proteins*.

	mLCN2 (NP_005555.2)	mNGALR (NP_065105.3)	mLRP2 (NP_004516.2)	mMC4R (NP_005903)
**hLCN2** **(NP_032517)**	62%			
**hNGALR** **(NP_001347335.1)**		94%		
**hLRP2** **(NP_001074557)**			77%	
**hMC4R** **(NP_058673.2)**				94%

* Pairwise sequence comparisons was done using the blastp routine from the National Library of Medicine [113] using the information provided under given accession numbers.

#### 6.4.1 Example 1: Affinity of LCN2 to MMP-9

Previous studies have shown that human LCN2 can form tight heterodimers with human MMP-9 in human neutrophils (3, 115). Contrarily, murine LCN2 lacks the corresponding cysteine and does not associate with MMP-9 (116). MMP-9 also termed Gelatinase A has the ability to degrade extracellular matrix components (e.g., collagen, laminin, elastin, fibronection) and dysregulation of MMP-9 is associated with various diseases (117). It is associated with acute and chronic inflammatory conditions by liberation of extracellular matrix-sequestered cytokines and growth factors, enabling influx of leukocytes in the inflamed tissue, and affecting the permeability of blood brain barrier (117). The binding of human LCN2 to human MMP-9 is mediated *via* a disulfide bridge between free cysteine107 (C107) of human LCN2 (cf. **Figure 1B**) and the cysteine of the Pro-Arg-Cys-Gly-Val sequence of MMP-9 that is located in the pro-peptide domain of MMP-9 and relevant for inhibition of its catalytic site (118, 119). The binding of LCN2 provokes an enzyme-activating effect in the regulation of inflammatory and pathophysiological responses of granulocytes in the physiological activation of MMPs (118). Therefore, heterodimer formation between both proteins should have fundamental importance in the outcome of inflammatory diseases. Furthermore, the complex of LCN2 and MMP-9 plays a crucial role in the modulation of the metastatic phenotype of cancer cells that promotes cancer cell invasion and metastasis through protection of MMP-9 degradation, inducing MMP-9 activation, and increasing the overall activity of MMP-9 (120). Finally, it is most likely that the covalent binding of LCN2 and MMP-9 will change the overall affinity to putative receptors. It is hard to believe that this important function of LCN2 should be limited to the human ortholog.

#### 6.4.2 Example 2: Posttranslational modifications

Another difference is the finding that mouse LCN2 is a PKCδ substrate and needs to be phosphorylated at T115 to be secreted from neutrophils (121). Although the surrounding residues of T115 are well-conserved between mice and humans, no reports are available demonstrating that phosphorylation at that side is necessary to mediate human LCN2 secretion from neutrophils or any other immune cell. Similarly, there are several reports demonstrating that human LCN2 is an *N*-glycosylated protein (122–124). However, the number of potential *N*-glycosylation sites is different in both species. It has been shown that bacterially expressed LCN2 has capacity to form complexes with the siderophore enterochelin and that *N*-glycosylation is not required for secretion of LCN2 into exosomes suggesting that this posttranslational modification might not be relevant for the activity of LCN2 (61, 124). However, it might be possible that glycosylation alters the affinity for receptors or binding partners or stability of this lipocalin. In mice it has been shown that the *in vivo N*-glycosylation pattern of LCN2 impacts the distribution within murine tissues (125). It is further well accepted that the structure of *N*-glycan is involved in the apical sorting of proteins in epithelial cells and that loss of *N*-glycan moieties might lead to shorter half-life (124, 125). Therefore, potential differences in posttranslational LCN2 glycosylation of murine and human LCN2 might provoke significant differences in tissue distribution and stability. Similarly, a different status of amidation in both mouse and human LCN2 might have different impact on the pathogenesis of various diseases such as inflammation, endothelial dysfunction, and hypertension (126). Consequently, high- (HMW) and low-molecular weight (LWM) species of LCN2 in mice and humans can result from different (unknown) posttranslational modifications. If these modifications are induced by similar mechanisms and provoke identical biological effects in mice and humans is still not known. Finally, experiments performed to study impact of LCN2 on cell death, mitochondrial functions, and other biological attributes could result in different findings when conducted with recombinant non-glycosylated or glycosylated LCN2. Moreover, experiments can lead to erroneous results if cells are treated with LCN2 originating from a different species differing in sequence or carrying different posttranslational modifications that will affect the activity to putative receptors.

### 6.5 LCN2 receptors in homeostasis and disease

Mice genetically disrupted for individual LCN2 receptors show severe phenotypes or die perinatally (44, 46–48), while mice lacking *Lcn2* are viable (15). The fact that disruption of a potential LCN2 receptor provokes a more severe phenotype is not surprising, because the proposed receptors are multi-ligand binding receptors associated to many other pathways. However, the ectopic overexpression of NGALR confers on cells the ability to take up iron or undergo apoptosis, dependent upon the iron content of LCN2 (13). This finding indicates that some of the LCN2-receptor functions can be separated from others and are independent from other receptors. Nevertheless, it must be critically noticed that NGALR compared to LRP2 has about 1000 fold higher affinity for LCN2 and overexpression of NGALR or addition of LCN2 in a setting of an experiment will also modify the binding of LRP2 to LCN2. So how can we be sure that the observed biological effect (e.g. induction of apoptosis) is mediated through the NGALR axis and not through the LRP2 axis?

Moreover, numerous human diseases are associated with alterations in LCN2 receptor expression. In particular, NGALR expression was shown to be modified in myeloproliferative neoplasm (MPN), colorectal cancer (CRC), esophageal squamous cell carcinoma (ESCC), glioma, clear cell renal cell carcinoma (ccRCC), glomerulonephritis, hepatocellular carcinoma (HCC), alcoholic hepatitis, obesity, psoriasis, gastric cancer, endometrial cancer, and many other disorders (**Table 3**). It is obvious that modified receptor expression should have (at least in part) the same effect as altered expression of its ligand, i.e. LCN2. However, if this is in fact the case is not known yet. To further assess the level of LCN2 receptors in the disease state, screening with a validated antibody of various healthy organ systems would be strongly recommended. Certainly, a comparison between different species such as mice and humans would be advisable to clearly localize the expression of the different receptors. Finally, modified NGALR expression in disease will inevitably cause changes in the amount of LCN2 that can be bound to LRP2. This will lead to modified activity of this receptor branch. Therefore, it will be difficult/challenging/complex/problematic to estimate if disease-associated effects are mediated by altered NGALR expression or alternatively by subsequent alterations in LRP2 signaling.

**Table 3:** Expression of NGALR in human specimen.

Organ/ Localization	Disease	Specimen	Detection methods	Key findings	Reference
**Blood**	MPN	Human PBMCs isolated from CML blast crisis patients + controls	RT-PCR	- *SLC22A17* was downregulated in PBMCs from CML blast crisis patients compared to PBMCs form normal donors	(13)
		Human PBMCs isolated from CML patients + controls	Flow cytometry, RT-PCR, Western blot	- SLC22A17 was stronger expressed in the untreated CML patients compared to normal PBMCs - *SLC22A17* level was higher before treatment but decreased after FCR treatment - Blocking of SLC22A17 induced death in SLC22A17^high^ CML cells but not in SLC22A17^low/-^ CLL cells	(127)
		PBMCs isolated from MPN patient^1^	RT-qPCR	- *SLC22A17* showed opposite expression to LCN2 in serial samples (prior or after IT) of PBMCs in MPN patients - High BCR-ABL transcript (sign of CML) levels are associated with low *SLC22A17*, but high LCN2 expression	(128)
**Colon**	CRC	FFPE tissues from CRC patients	IHC	- SLC22A17 was increased in CRC compared to normal adjacent glandular tissue - Upregulated SLC22A17 was linked with deeper invasion and high degree of TNM - SLC22A17/LCN2 correlates with deeper invasion, tumor progression and co-expression with Ki67 and ferritin	(129)
**Oesophagus**	ESCC	Frozen ESCC tissues	RT-PCR	- *SLC22A17* has three splice variants - *SLC22A17* variant 3 was increased in 70% of ESCC tissues compared with normal adjacent tissue - *SLC22A17* variants 1 and 2 were upregulated in 55% of ESCC tissues	(50)
		Frozen ESCC tissue	RT-PCR, IHC	- SLC22A17 increased in ESCC tissue compared to normal epithelium on protein and mRNA level	(130)
		FFPE ESCC tissue	Tissue microarray/IHC	- Increase in SLC22A17/LCN2 positively correlated with poor prognosis - High SLC22A17 connected with lower 5-year survival rate in ESCC patients	(131)
**Head/brain**	Glioma	FFPE glioma specimen (for staining), frozen glioma specimen (for Western blots and RT-qPCR) + controls	IHC, Western blot, RT-qPCR	- SLC22A17/LCN2 are higher in glioma tissues but rarely present in non-neoplastic brain tissues (RNA and protein) - Overexpression of SLC22A17/LCN2 in glioma patients correlate with poor prognosis and low survival	(132)
	AD	Frozen brain tissue from patients with AD + controls	Western blot	- No differences in SLC22A17 expression between healthy controls and patients with AD in nine different brain regions - No correlation of LCN2 with SLC22A17	(133)
	LM	CSF from cancer patients (isolated by lumbar puncture, cistern, or Ommaya tap)	Single-cell sequencing, immunofluorescence	- Cancer cells within the CSF express *SLC22A17* mRNA and protein (and LCN2)	(134)
**Kidney**	ccRCC	ccRCC tissue data sets from data bases + controls	Data analysis	- *LCN2*/*SLC22A17* were downregulated in ccRCC samples in most of the data sets	(135)
	Glomerulonephritis	FFPE tissues of glomerulonephritis patients + controls	IHC	- SLC22A17 was high in glomerulonephritis patients	(136)
**Liver**	HCC	FFPE tumor tissues from curative resection of HCC	IHC	- SLC22A17 (and LCN2) were increased in HCC tissues compared with adjacent non-tumorous liver - SLC22A17 (and LCN2) correlate with vascular invasion, TNM stage, recurrence, poor prognosis and shorter overall survival	(137)
	AH	Tissue from AH patients (different disease stage) + controls	IF, RT-qPCR	- SLC22A17 was slightly increased in liver tissue of AH patients	(103)
**Metabolism**	Obesity	GWAS	Data analysis	- *SLC22A17* was associated with BMI and BMR in obese Korean woman	(138)
		Metabolic GWAS	Data analysis	- *SLC22A17* was associated with different metabolites in obese Korean woman	(139)
		GWAS	Data analysis	- *SLC22A17* was inversely associated with a new SNP that was linked to Japanese food score in a Japanese population	(140)
	ATC	DNA isolated from specimen of childhood cancer patients (blood, salvia or buccal swabs)^2^	Genotyping, data analysis	- A novel SNP of *SLC22A17* (risk variants) was identified which is associated with ACT	(141)
**Pancreas**	PDAC	Gene expression data, clinical information	Data analysis	- *SLC22A17* identified as a TMB-related immune gene and high expression correlated with better prognosis of PDAC patients	(142)
**Skin**	Psoriasis	Neutrophils isolated from peripheral blood (of psoriatic patients) + controls	RT-PCR, IF	- *SLC22A17* expression was higher in neutrophils from psoriatic patients than control - SLC22A17 was expressed on the surface of neutrophils and identified in lesional dermis	(143)
		Skin biopsies of patients with psoriasis + control skin	RT-qPCR	- *SLC22A17* was increased in lesional epidermis of patients with psoriasis	(144)
**Stomach**	GC	Gene expression data from database, FFPE GC tissue	Data analysis, IHC	- High *SLC22A17* expression was found as a prognostic marker for gastric cancer, associated with infiltration of immune cells and poor overall survival - SLC22A17 was increased in GC samples compared with normal adjacent tissue	(145)
		Gene expression data from database	Data analysis	- Correlation analysis identified *SLC22A17* as a hub gene in GC, associated with worse overall survival	(146)
	(advanced) GC	Gene expression data from database	Data analysis	- *SLC22A17* considered as prognostic biomarker in GC	(147)
**Uterus**	EC	FFPE tissue from EC	IHC	- SLC22A17 expression was enhanced in EC tissue compared with no expression in normal adjacent tissue - In apex of papillary structures co-expression of LCN2 and SLC22A17 was found - Increased SLC22A17 expression was associated with higher tumor grade, advanced tumors and lower survival rate	(148)
	Preeclampsia	Placenta tissue with different severity of preeclampsia^3^	RT-PCR, Western blot	- No difference in *SLC22A17* mRNA or protein was found in woman with different severity of preclampsia (in contrast to *LCN2*)	(149)

^1^ one patient in different stages of MPN disease (before and after imatinib treatment); ^2^ two different cohorts of patients were genotyped for 4578 SNPs to capture genetic variation of various key drug biotransformation genes; ^3^ only English abstract available; paper in Chinese; AD, Alzheimer’s disease; AH, alcoholic hepatitis; ATC, anthracycline-induced cardiotoxicity; BMI, body mass index; BMR, basal metabolic rate; ccRCC, clear cell renal cell carcinoma; CLL, chronic lymphocytic leukemia; CML, chronic myelogenous leukemia; CRC, colorectal cancer; CSF, cerebrospinal fluid; EC, endometrial cancer; ESCC, esophageal squamous cell carcinoma; FCR, fludarabine, cyclophosphamide and rituximab; FFPE, formalin-fixed paraffin-embedded; GC, gastric cancer; GWAS, genome-wide association study; HCC, hepatocellular carcinoma; IF, immunofluorescence; IHC, immunohistochemistry; IT, imatinib; LM, leptomeningeal metastasis; MPN, myeloproliferative neoplasms; PBMCs, peripheral blood mononuclear cells; PDAC, pancreatic ductal adenocarcinoma; RT-PCR, reverse-transcriptase PCR; RT-qPCR, reverse-transcriptase quantitative real-time PCR; SNP, single nucleotide polymorphisms; TMB, tumor mutational burden; TNM, tumor, node and metastasis.

## 7 Conclusions

LCN2 is a pleiotropic molecule involved in many biological processes including control of iron homeostasis, cell differentiation, energy expenditure, bacterial defense, cell death, chemotaxis, cell migration, and many others. Most of the biological functions of LCN2 were established in work conducted in mice. Nowadays, six potential LCN2 receptors (NGALR, LRP2, LRP6, MCR4, MCR1, and MCR3) were identified that markedly differ in regard to structure and affinity. In humans, concentrations of LCN2 in urine and serum have become increasingly relevant as a prognostic and predictive biomarker for many diseases (150, 151). Nevertheless, changes in LCN2 or LCN2 receptor expression in the course of a specific disease was mostly only reported in association or correlation studies. Actually, there is a significant gap in the understanding of the underlying mechanisms by which LCN2 and its putative receptors contribute to the initiation or progression of a disease. It could be that some findings found in experimental mouse models cannot be translated 1:1 to the human situation. Murine and human LCN2 only share a sequence identity of 62% and there are significant biochemical and functional differences between both orthologs. This might result in different affinities for putative receptors and other biomolecules supposed to interact with LCN2, resulting in modified biological effects in both species. Contradictory findings with regard to biological effects of LCN2 show that the effects of LCN2 are complex and influenced by many factors, including species, disease, sex, cell type, posttranslational modifications, receptors, and binding partners. Possibly, some of the discrepancies and inconsistencies are raised by the fact that many findings on LCN2 and its receptors were established in *in vitro* models and have no relevance for the *in vitro* situation. Finally, the usage of antibodies with unclear sensitivity and specificity may lead to data that cannot be interpreted. It cannot be excluded that incorrect expression patterns of the LCN2 receptors can also be found in the literature because the antibodies used have not been sufficiently validated. We hope that our article will help to sensitize scientists for this critical issue that may provoke the production of misleading “scientific” data.

## Author contributions

RW conceived and planned the drafting of this review. All other authors have written part of this review and helped in design and drawing of images. All authors contributed to the article and approved the submitted version.

## Glossary (in alphabetical order)

**Table:**

α-MSH	α-melanocyte-stimulating hormone
α_1_M	α_1_-microglobulin
ACTH	adrenocorticotropin hormone
ACKR3	atypical chemokine receptor 3
APOM	apoproteinM
BMP-4	bone morphogenetic protein-4
BOCT	brain type organic cation transporter
cAMP	cyclic adenosine-monophosphate
CRC	colorectal cancer
CXCR7	CXC motif receptor 7
EGF	epidermal growth factor
ERK	extracellular regulated kinase
ESCC	esophageal squamous cell carcinoma
FGF	fibroblast growth factor
GPR159	G protein-coupled receptor 159
HCC	hepatocellular carcinoma (HCC)
LCN2	Lipocalin 2
LRP2	lipoprotein-related protein 2
MC4R	melanocortin 4 receptor
MFS	major facilitator superfamily
MMP	metalloproteinase
MPN	Myeloproliferative neoplasm
MUP6	major urinary protein 6
NGAL	neutrophil gelatinase-associated lipocalin
NGALR	neutrophil gelatinase-associated lipocalin receptor
NMR	nucleic magnetic resonance
NRL	neu-related lipocalin
OBPIA	odorant-binding protein IA
PKC	kinase protein kinase C
PLC	phospholipase C
PrEST	protein epitope signature tag
RBP	retinol binding protein
Scn	siderocalin
SIP24	super inducible protein 24
SLC	solute carrier
SLC22A17	solute carrier family 22 member 17
SPR	surface plasmon resonance
SSH	sonic hedgehog signaling
TNF-α	tumor necrosis factor-α
