# Supplementary material for: Lipocalin 2 receptors: facts, fictions, and myths
Source: Front Immunol. 2023 Aug 11;14:1229885. doi: 10.3389/fimmu.2023.1229885 (PMC10451079; doi:10.3389/fimmu.2023.1229885)
Supplement: Supplementary file 1 [file Table_1.docx]

Supplementary Material

Lipocalin 2 receptors: Facts, Fictions, and Myths

Sabine Weiskirchen, Ralf Weiskirchen^*^

*** Correspondence:** Corresponding Author: rweiskirchen@ukaachen.de

# Supplementary Figures and Tables

# Suppl. Table 1: List of abbreviations for renal cell subsets used in Figure 4

| **Abbreviation** | **Cluster** |
| --- | --- |
| aFIB | Fibroblast (adaptive / maladaptive / repairing^1^) |
| aPT | Proximal Tubule Epithelial Cell (adaptive / maladaptive / repairing^1^) |
| aTAL1 | Thick Ascending Limb Cell Cluster 1 (adaptive / maladaptive / repairing^1^) |
| aTAL2 | Thick Ascending Limb Cell Cluster 2 (adaptive / maladaptive / repairing^1^) |
| B | B Cell |
| C-TAL | Cortical Thick Ascending Limb Cell |
| cDC | Classical Dendritic Cell |
| CNT | Connecting Tubule Cell |
| CNT-IC-A | Connecting Tubule Intercalated Cell Type A |
| CNT-PC | Connecting Tubule Principal Cell |
| cycEC | Endothelial Cell (cycling^2^) |
| cycEPI | Epithelial Cell (cycling^2^) |
| cycMNP | Mononuclear Phagocyte (cycling^2^) |
| cycT | T Cell (cycling^2^) |
| dC-TAL | Cortical Thick Ascending Limb Cell (degenerative^3^) |
| dCNT | Connecting Tubule Cell (degenerative^3^) |
| dCNT-PC | Connecting Tubule Principal Cell (degenerative^3^) |
| DCT1 | Distal Convoluted Tubule Cell Type 1 |
| dDCT | Distal Convoluted Tubule Cell (degenerative^3^) |
| dEC-PTC | Peritubular Capillary Endothelial Cell (degenerative^3^) |
| dIC-A | Intercalated Cell Type A (degenerative^3^) |
| dPC | Principal Cell (degenerative^3^) |
| dPT | Proximal Tubule Epithelial Cell (degenerative^3^) |
| dPT/DTL | Proximal Tubule Epithelial Cell / Descending Thin Limb Cell (degenerative3) |
| DTL1 | Descending Thin Limb Cell Type 1 |
| dVSMC | Vascular Smooth Muscle Cell (degenerative^3^) |
| EC-AEA | Afferent / Efferent Arteriole Endothelial Cell |
| EC-GC | Glomerular Capillary Endothelial Cell |
| EC-LYM | Lymphatic Endothelial Cell |
| EC-PTC | Peritubular Capillary Endothelial Cell |
| FIB | Fibroblast |
| IC-A | Intercalated Cell Type A |
| IC-B | Intercalated Cell Type B |
| M-TAL | Medullary Thick Ascending Limb Cell |
| MAC-M2 | M2-Macrophage |
| MAST | Mast Cell |
| MC | Mesangial Cell |
| MDC | Monocyte-derived Cell |
| MON | Monocyte |
| MyoF | Myofibroblast |
| ncMON | Non-classical Monocyte |
| NK1 | Natural Killer Cell Type 1 |
| NK2 | Natural Killer Cell Type 2 |
| NKT | Natural Killer T Cell |
| PC | Principal Cell |
| pDC | Plasmacytoid Dendritic Cell |
| PEC | Parietal Epithelial Cell |
| PL | Plasma Cell |
| POD | Podocyte |
| PT-S1/S2 | Proximal Tubule Epithelial Cell Segment 1 / Segment 2 |
| PT-S3 | Proximal Tubule Epithelial Cell Segment 3 |
| REN | Renin-positive Juxtaglomerular Granular Cell |
| T | T Cell |
| T-CYT | Cytotoxic T Cell |
| T-REG | Regulatory T Cell |
| tPC-IC | Principal-Intercalated Cell (transitional^4^) |
| VSMC/P | Vascular Smooth Muscle Cell / Pericyte |

**Footnotes:** ^1^adaptive/maladaptive/repairing: Represented by cells that retain differentiation markers of reference states, albeit at lower levels, but also show expression of known injury associated genes, mesenchymal markers or factors promoting inflammation or fibrosis. ^2^cycling: Represented by enrichment of cell cycle genes. ^3^degenerative: Marked loss of differentiation markers, and/or increased %ERT, %MT, and/or marked decrease in genes detected. These cells could represent an early injury state or cells that will not recover function. ^4^transitional: Represented by an intermediate state showing markers of cells sharing the same parental lineage. The list was taken from: [https://atlas.kpmp.org](https://eur02.safelinks.protection.outlook.com/?url=https%3A%2F%2Fatlas.kpmp.org%2F&data=05%7C01%7C%7C2ccfc32e2bab4d5d505208da70430eba%7C5a6d5ee56edf4a26ba93f5872dbb9614%7C0%7C0%7C637945730577603931%7CUnknown%7CTWFpbGZsb3d8eyJWIjoiMC4wLjAwMDAiLCJQIjoiV2luMzIiLCJBTiI6Ik1haWwiLCJXVCI6Mn0%3D%7C3000%7C%7C%7C&sdata=H12ZsewK7E34i3RXswJettOTjGMrWyBCcVnh%2BZJDlS0%3D&reserved=0).

**
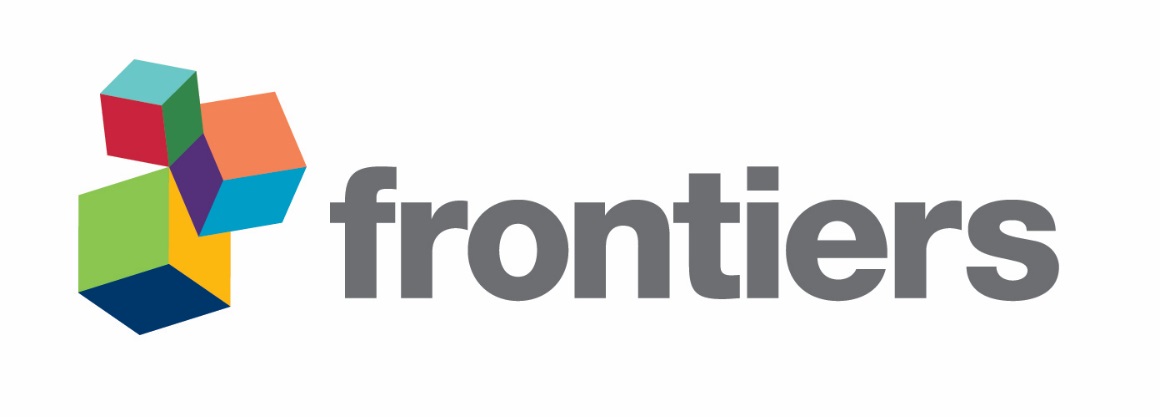
**
